# RpoS activates *Salmonella* Typhi biofilms and drives persistence in a Zebrafish model

**DOI:** 10.1101/2023.10.26.564249

**Published:** 2023-10-26

**Authors:** Stuti K. Desai, Yiyang Zhou, Rahul Dilawari, Andrew L. Routh, Vsevolod Popov, Linda J. Kenney

**Affiliations:** 1 Department of Biochemistry and Molecular Biology, University of Texas Medical Branch, Galveston, TX 77555; 2 Sealy Center for Structural Biology and Molecular Biophysics, University of Texas Medical Branch, Galveston, TX 77555; 3 Institute for Human Infections and Immunity, University of Texas Medical Branch, Galveston, TX 77555; 4 Department of Pathology, University of Texas Medical Branch, Galveston, TX 77555; 5 Department of Immunology and Microbiology, Scripps Research, 10550 N. Torrey Pines Rd., La Jolla, CA 92037, USA

**Keywords:** Static immersions, Transposon directed insertion site sequencing, ClickSeq, Asymptomatic Typhoid carriage, IraP, Fimbriae, Vi-antigen, heterologous host, Zebrafish

## Abstract

The development of strategies for targeting the asymptomatic carriage of *Salmonella* Typhi in chronic typhoid patients has suffered owing to our basic lack of understanding of the molecular mechanisms that enable the formation of *S.* Typhi biofilms. Traditionally, studies have relied on cholesterol-attached biofilms formed by a closely related serovar, Typhimurium, to mimic multicellular Typhi communities formed on human gallstones. In long-term infections, *S.* Typhi adopts the biofilm lifestyle to persist in vivo and survive in the carrier state, ultimately leading to the spread of infections via the fecal-oral route of transmission. In the present work, we studied *S.* Typhi biofilms directly, applied targeted as well as genome-wide genetic approaches to uncover unique biofilm components that do not conform to the CsgD-dependent pathway as established in *S.* Typhimurium. We adopted a genome-wide *Tn5* mutation screen in *S.* Typhi in gallstone-mimicking conditions and generated New Generation Sequencing libraries based on the ClickSeq technology to identify the key regulators, IraP and RpoS, and the matrix components as Sth fimbriae, Vi capsule and lipopolysaccharide. We discovered that the starvation sigma factor, RpoS, was required for the transcriptional activation of matrix-encoding genes in vitro, and for *S.* Typhi colonization in persistent infections in vivo, using a heterologous fish larval model. Overall, our work established a novel RpoS-driven paradigm for the formation of cholesterol-attached Typhi biofilms and emphasized the role(s) of stress signaling pathways for adaptation in chronic infections.

## Introduction

*Salmonella enterica* is a rod-shaped enteric bacterium that easily spreads through contaminated food or water via the fecal-oral route in poor hygiene conditions. The human-restricted serovar of *Salmonella enterica*, serovar Typhi (STy), causes typhoid fever and continues to be a dangerous pathogen throughout the world^[1]^. The global incidence of typhoid fever in mostly children, adolescents and older adults is between 12 to 27 million and in total 116,815 succumbed to the disease in 2017^[2]^. In contrast, its closely related serovar Typhimurium (STm) infects diverse hosts such as humans, cattle, poultry and reptiles to cause gastroenteritis that is mostly self-limiting in healthy adults.

Upon successful invasion of intestinal epithelial cells, *Salmonella* is phagocytosed by macrophages, where it resides in a modified vacuole in a self-nourishing niche called a *S**almonella*-Containing Vacuole (SCV) to ultimately reach the systemic sites of liver, spleen and the gall bladder. The SsrA/B Two-Component Regulatory System (TCRS) is essential for the activation of the *S**almonella* Pathogenicity Island-2 (SPI-2) regulon genes encoding a type-three secretory system and effectors that are involved in formation of the SCV^[3–5]^. *Salmonella* also resides encased in extracellular matrix as multicellular communities or biofilms, on intestinal epithelial cells^[6]^, gallstones^[7]^, tumors^[8]^ and in the large intestine^[9]^.

Interestingly, SsrB, a response regulator of the SsrA/B TCRS, is essential for switching on the multicellular lifestyle of *S.* Typhimurium by relieving H-NS silencing at the *csgD* promoter^[10, 11]^. CsgD is the master regulator of STm biofilms, and it activates the transcription of extracellular matrix components including curli fimbriae, cellulose, BapA and the O-antigen capsule^[12–16]^. Moreover, the SsrB-CsgD regulatory pathway drives STm persistence in the heterologous host *Caenorhabditis elegans* by enabling the formation of biofilms, which eventually promotes host life span through the p38-Mitogen-activated Protein Kinase (p38-MAPK) innate immunity pathway^[17]^.

Biofilms in the gall bladder are important for maintaining the carrier state of *Salmonella* Typhi, allowing it to persist in 2 to 4% of chronic typhoid patients^[18–20]^. STy reservoirs in human carriers, who are characteristically asymptomatic, play a crucial role in the spread of typhoid in epidemic regions, as well as in its introduction to non-epidemic regions. Indeed, such long-term colonization of STy in human carriers, coupled with the rise of multi-drug resistant strains, for example, those belonging to the H58 haplotype, prevents effective control of typhoid fever ^[21–23]^. Unfortunately, all of our understanding of the regulation of STy biofilms on gallstones has been based on the assumption that it shares a high degree of conservation with canonical biofilms formed by the closely related serovar, *S.* Typhimurium.

Herein, we establish that STy biofilms are fundamentally distinct from STm biofilms. In particular, CsgD and the STm lifestyle regulator, SsrB, are not required for formation of multicellular aggregates on surfaces coated with cholesterol that forms the major component of human gallstones. In order to identify unique components of STy biofilms, we employed a whole genome transposon mutagenesis approach, Tn-ClickSeq, and identified the starvation sigma factor RpoS as a crucial determinant of STy biofilms. We discovered that the formation of large STy aggregates was defective in the absence of *rpoS* owing to the down-regulation of extracellular matrix components comprised of Sth fimbriae, the Vi-antigen and the lipopolysaccharide core.

Finally, we developed a heterologous host model, *Danio rerio*, to investigate STy lifestyles in persistent infections for visualizing the colonization in real-time by confocal microscopy and measuring the ensuing effects on host physiology. Zebrafish is a powerful vertebrate model for many human diseases owing to a high degree of conservation of immuno-signaling pathways and colonization characteristics of bacterial infections^[24–27]^. Previous studies have established that exposure of zebrafish larvae to *S.* Typhimurium, *Mycobacterium marinum*, *Shigella flexneri*, and *Pseudomonas aeruginosa* leads to successful pathogenesis^[28–32]^. *Shigella sonnei* and *S.* Typhimurium have also been recently shown to persistently colonize macrophages in zebrafish^[33, 34]^. We infected zebrafish larvae with STy using static immersions. Intestinal colonization was diminished in the *rpoS* null and larval survival at 6 days post infection was greater, corroborating a crucial role of RpoS in prolonged STy survival in vivo.

## Results

### *S.* Typhi biofilms employ unique components that differ from *S.* Typhimurium biofilm

**Figure 1:**
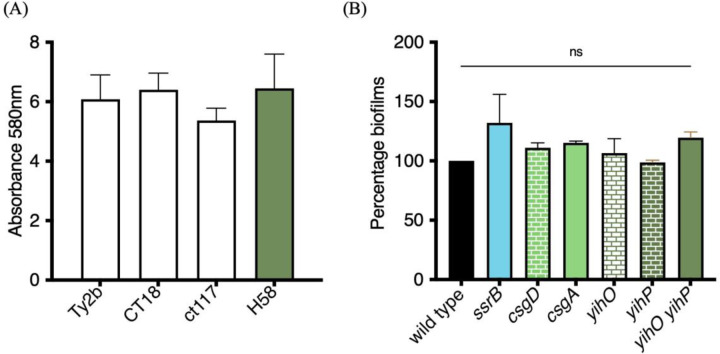
The master regulator CsgD, and the STm lifestyle regulator SsrB, are not required for the formation of cholesterol-attached STy biofilms. Crystal violet staining assays: (A) Wild type strains Ty2b, CT18, ct117 and H58 formed robust biofilms at day 2 in gallstone-inducing conditions and (B) STy null strains deleted for the indicated STm-biofilm homologs formed similar biofilms as the H58 wild type strain at day 2. Growth medium added to cholesterol-coated Eppendorf tubes was used as the control and subtracted from all measurements. N = 3 in at least triplicates, error bars represent Mean ± SD, ns = not significant by One-way ANOVA.

Our previous analysis of *S.* Typhimurium (STm) biofilms established that unphosphorylated SsrB was required for the expression of the master biofilm regulator CsgD to drive biofilm formation in vitro and in vivo in the intestinal lumen of *C. elegans*^[10, 35]^. *S.* Typhi (STy) forms biofilms on gallstones in the gall bladder and this ability is an important aspect of maintaining its carrier state^[18–20]^. It was therefore of interest to examine whether STy employed similar pathways as STm for biofilm formation. We grew the STy strain H58 under conditions that were proposed to mimic the gall bladder environment in humans^[36]^, hereinafter referred to as gallstone-mimicking conditions, and observed robust surface-attached communities after two days in cholesterol-coated tubes, as measured by a crystal violet staining assay (Fig. 1A). The requirement of such distinct physico-chemical factors to develop biofilms was not specific to H58, as three other isolates including Ty2b, CT18 and ct118 formed comparable cholesterol-attached biomass after two days of growth (Fig. 1A). Not surprisingly, H58 also failed to form the characteristic rough, dry and red (‘rdar’) morphotype at 30°C and low osmolality, as classically observed for the STm wild type 14028s (Supplementary Fig. 1A). The loss of the rdar morphotype, as well as the inability to form biofilms in STm-specific environmental conditions has also been recently described for six other STy wild type strains^[37]^. We followed the developmental course of H58 biofilm formation at two, four and six days in cholesterol-coated tubes and observed only a marginal increase in the amount of biofilms at days 4 and 6 compared to day 2 (Supplementary Fig. 1B). We therefore focused on understanding STy biofilms at day 2 in our further investigations.

The response regulator SsrB plays a dual role in activating the virulent lifestyle in *S.* Typhimurium (SsrB~P-dependent activation of SPI-2 genes), while driving the formation of biofilms in its unphosphorylated form by de-repressing *csgD*^[10, 11 for a review]^. Hence, we examined whether complete deletions of *ssrB* and *csgD* affected the ability to form cholesterol-attached *S.* Typhi biofilms. Interestingly, biofilm formation was not affected by the loss of STy homologs encoding either the STm lifestyle regulator SsrB, or the master regulator of STm biofilms CsgD, emphasizing the fundamental differences in mechanisms of biofilm formation in these two closely related serovars (Fig. 1B). We also investigated whether adhesion of STy aggregates to cholesterol-coated surfaces required curli fibers and O-Antigen, as observed in the STm model, by inactivating *csgA* and *yihO*/*P,* respectively^[13, 38]^. We discovered that curli fibers and O-Antigen did not play any role in the formation of cholesterol-attached STy biofilms, strongly establishing that STy biofilms are drastically different from the model STm biofilms (Fig. 1B).

### Tn-ClickSeq reveals the unique genetic signature of STy biofilms

**Figure 2:**
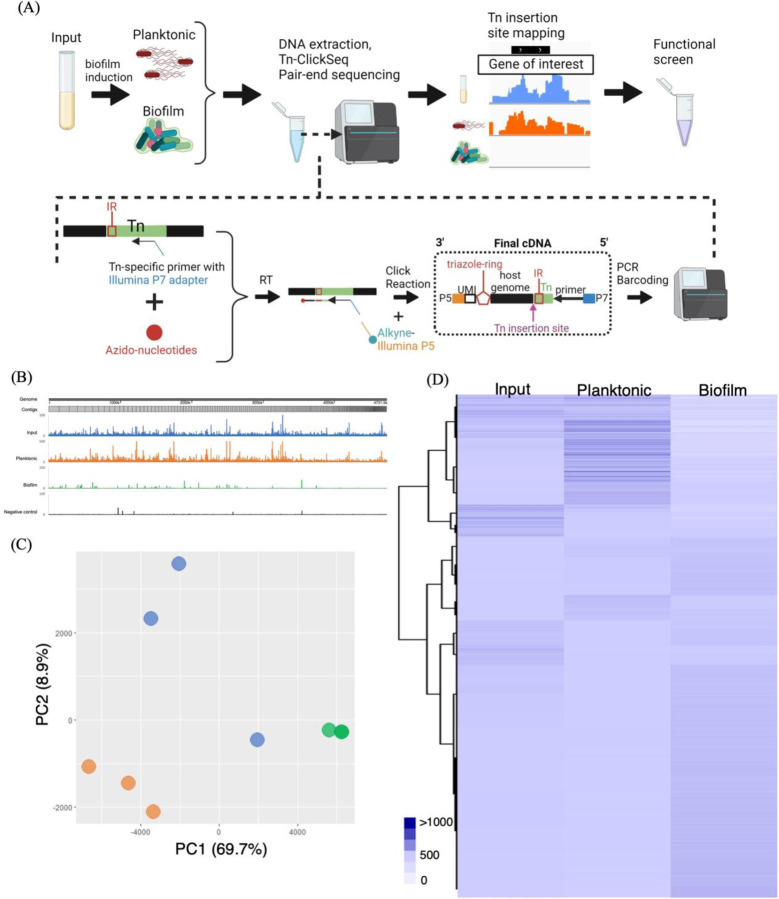
Tn-ClickSeq analysis investigated novel pathways that drive the formation of STy biofilms. (A) A general scheme depicting Tn-ClickSeq analysis, from the isolation of planktonic and biofilm fractions, DNA extraction and the generation of Tn-ClickSeq libraries using a reverse transcription reaction (RT) with a Tn-specific primer and azido-nucleotides/dNTP mixtures. The azido-terminated cDNAs were click-ligated to an alkyne-adapted illumina P5 adapter to generate Illumina libraries. After PCR and barcoding, the reads that contained partial Tn sequence and genome insertion site were pair-end sequenced and subjected to downstream analysis. (B) A linearized map of the average Tn-insertion sites from three biological replicates showed genome-wide differences in the planktonic sub-population (orange) and biofilms (green). The H58-Tn library inoculum (blue) and the H58 parent without any transposons (black, control) were used as positive and negative controls, respectively. (C) Principal component analysis showed distinct clustering of respective replicates of planktonic (orange) and biofilm (green) fractions as compared to the input library (blue). (D) The number of transposon insertions of each gene was normalized to per 1000 bp of gene length and averaged among replicates for each group to depict a hierarchical clustering of Tn-ClickSeq targets.

Transposon-directed insertion site sequencing (TraDIS) has been immensely useful to study the genetic repertoires essential for growth in STy and *Escherichia coli*, evolution of the invasive STm lineage ST313, and biofilm formation in *E. coli* and *Pseudomonas aeruginosa*^[39–43]^. We applied a similar approach, Tn-ClickSeq, by combining genome-wide transposon (Tn) mutagenesis with ClickSeq. ClickSeq is advantageous here since it is a fragmentation-free next-generation sequencing (NGS) library synthesis technique and is capable of generating focused NGS read data upstream of chosen target sites^[44, 45]^, thus greatly simplifying the digital transposon display protocol and removing artefactual recombination events inherent to common NGS library preparation techniques. Using primers targeting the 3’ or 5’ ends of inserted transposons, Tn-ClickSeq can sensitively and specifically sequence the junctions of a transposon and the adjacent genomic loci of the integration site and thus identify genetic loci involved in forming cholesterol-attached biofilms in vitro (Fig. 2A). A Tn-library in the *S.* Typhi strain H58 was kindly provided by Stephen Baker, Cambridge University, UK^[46]^. We grew *S.* Typhi biofilms for two days using the H58-Tn library in gallstone-mimicking conditions and isolated planktonic and biofilm fractions from a pool of thirty cholesterol-coated tubes. NSG libraries were generated from genomic DNA isolated from each of these sub-populations using the Tn-Click-Seq approach^[45]^ (Fig. 2A).

Since Tn-ClickSeq returns short sequence reads containing a short fragment of the 3’ or 5’ end of the inserted (known) Tn as well as a fragment of the adjacent genomic DNA, we developed a simple computational pipeline that identified and trimmed the Tn-derived sequences from individual reads, and then mapped the remaining fragment to the H58 genome (SAMEA3110714) using HISAT2. The 3’ end of these Tn-mapping reads represent the exact nucleotide junction of the insertion site of the Tn in the genome. With this approach, we identified transposon insertions as 47%, 52% and 20% per million raw reads in the input, planktonic and biofilm sub-populations, respectively (Supplementary Fig. 2A and B). Mapping of insertion sites returned genomic locations of the inserted Tn in each dataset as well as the frequency of these inserts within each original sample. After controlling for PCR duplication using UMIs included in the ‘Click-Adaptor’^[44]^, we assigned insertion indices by dividing the number of Tn-insertions per gene with 1 Kbp of gene length (TnClickSeq insertion indices.xlsx). With this approach, gene insertion frequencies in each condition revealed clear differences in the planktonic and biofilm fractions, indicating unique genetic components driving H58 lifestyles in gallstone-mimicking conditions (Fig. 2B). Principal Component Analysis (Fig. 2C) and hierarchical clustering analysis (Fig. 2D) returned distinct clusters of genome insertions sites of the three replicates of Tn-ClickSeq libraries obtained from the input, planktonic and biofilm sub-populations. Overall, we successfully adopted the Tn-ClickSeq approach in *S.* Typhi to generate a comprehensive view of genetic systems that determine the development of cholesterol-attached biofilms from unattached planktonic cells.

### Identifying matrix components and regulators of STy biofilms

To determine the exact mechanism(s) by which STy forms multicellular communities on cholesterol surfaces in gallstone-mimicking conditions, we compared insertion indices between the planktonic and biofilm fractions. Transposon insertions in biofilm genes likely disrupt functions resulting in the inability of such mutants to form biofilms and would be enriched in the planktonic sub-population. Interestingly, the majority of insertion indices had drastically lower values in the biofilm fraction compared to the planktonic group, and these were of immediate interest for validation as STy biofilm targets (TnClickSeq insertion indices.xlsx). We focused on loci having mean insertion indices greater than 100 in the planktonic sub-population and performed Gene Ontology analysis on 1,515 such genes and identified a significant enrichment in cell membrane components, transmembrane ion transport pathways, and other membrane-related activities (Supplementary Fig. 2C). However, it was not possible to perform essentiality analysis using a standard bioinformatics pipeline, because most of these genes had insertion indices equal to zero in the biofilm fraction (see Methods and Discussion). In the absence of any *a priori* list of ‘essential’ biofilm components, we narrowed our list of 1,515 genes to the top 200 and sought to investigate some for their roles in STy biofilms. It was noteworthy that genes/operons encoding STm ‘biofilm’ homologs; *ssrB*, *csgDEFG*, *csgBAC* or *yihPO* did not appear on our list of selected Tn-ClickSeq targets. This established a strong correlation of our targeted genetic approach as described in Figure 1B, to our whole genome transposon mutagenesis Tn-ClickSeq approach (Fig. 2A).

**Figure 3:**
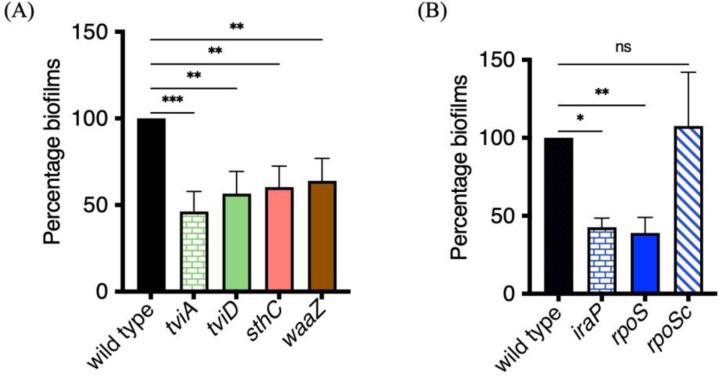
Biofilms are defective in STy null mutants identified by Tn-ClickSeq. (A) Defective biofilms formed by *tviA/tviD*, *sthC* and *waaZ* null strains suggested a role of Vi capsule, Sth fimbriae and the lipopolysaccharide core in extracellular matrix production, respectively. (B) H58 strains deleted for *iraP*, which regulates the protein stability of RpoS, and *rpoS,* which encodes the starvation sigma factor, formed less biofilms than the wild type parent. The defect in biofilm formation of the *rpoS* null strain was complemented by overexpression of *rpoS* from a plasmid *in trans*. N = 3, in at least triplicates, error bars represent Mean ± SD, in a crystal violet staining assay. Growth medium added to cholesterol-coated Eppendorf tubes was used as the control and subtracted from all measurements, ns = not significant, *p ≤ 0.05, **p ≤ 0.01 and ***p ≤ 0.001 by one-way ANOVA.

We next generated precise deletions of selected loci identified by Tn-ClickSeq that might encode the structural components of STy biofilms in our gallstone-mimicking conditions: *sthC*, part of the *sth* fimbrial operon^[47, 48]^, *waaZ*, encoding an enzyme involved in the biosynthesis of the LPS core forming the outer membrane^[49]^ and *tviD*, a part of the *tviBCDE* operon encoding for the surface-exposed Vi-Antigen; TviA is the regulator^[50, 51]^. We performed crystal violet staining assays at day 2 and determined that the loss of *sthC*, *waaZ, tviA* and *tviD* led to a decrease in *S.* Typhi biofilms (Fig. 3A).

In parallel, while unraveling the regulatory circuits that drive the formation of surface-attached STy communities, we were intrigued by the gene *iraP*, as it encodes an anti-adapter that inhibits the ClpXP-dependent proteolysis of the starvation master regulator RpoS^[52]^. We tested the biofilm formation capabilities of an *iraP* null H58 strain and observed a substantial reduction in biofilms compared to the wild type parent at 2 days (Fig. 3B). Since the only known role of IraP is an indirect positive role in activating the starvation stress response in enteric bacteria^[53]^, we examined whether deletion of *rpoS* affected *S.* Typhi biofilms. Indeed, we discovered a positive effect of RpoS in regulating the formation of cholesterol-attached STy biofilms (Fig. 3B). Over-expression of RpoS *in trans* (a kind gift from Roy Curtiss III, University of Florida), complemented the defect in biofilm formation of the *rpoS* null strain (Fig. 3B) and increased biofilms compared to the wildtype. Our discovery of RpoS as an activator of *S.* Typhi biofilms is significant, as biofilms were not mediated through CsgD (unlike in *S.* Typhimurium, see Fig. 1B). Finally, we ruled out an effect of mere growth differences on biofilm formation in the STy mutants by monitoring the planktonic growth of *iraP*, *rpoS*, *sthC*, *waaZ* and *tviD* null mutants compared to the wild type H58 parent. We observed similar growth rates among all the strains indicating that IraP, RpoS, SthC, WaaZ and TviD were necessary for the formation of *S.* Typhi surface-attached communities (Supplementary Fig. 3).

### Cholesterol-attached aggregates of STy biofilm mutants exhibit a poor ultrastructure

**Figure 4:**
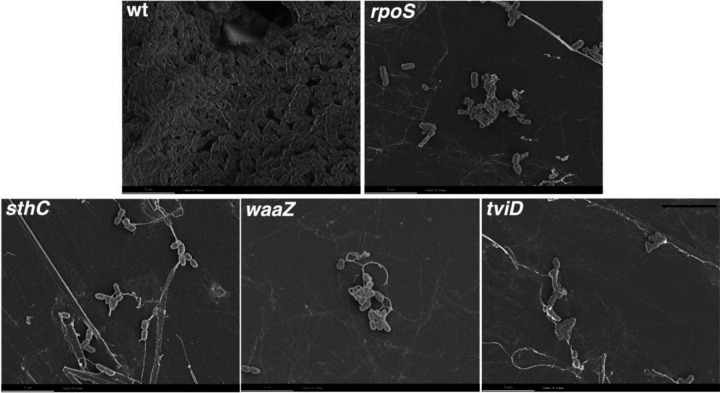
Ultra-structure visualization reveals the loss of dense aggregate formation in STy biofilm mutants. Representative scanning electron microscopy images of rich *S.* Typhi biofilms formed by the H58 (wt) and strikingly smaller sized aggregates formed by the strains deleted of *rpoS*, *sthC*, *waaZ* or *tviD* when grown in cholesterol-coated Eppendorf tubes in gallstone-mimicking conditions. Scale bar = 5 μm.

Our understanding of the CsgD-dependent mechanisms of biofilm formation in *S.* Typhimurium has been achieved by screening mutant strains for their inability to exhibit the rdar morphotype and high-resolution imaging of biofilm components by fluorescence confocal microscopy^[10, 35, 37, 54–56]^. Unfortunately, neither of these approaches can be applied to unequivocally establish the molecular players that drive the formation of STy biofilms on cholesterol-coated surfaces because of a high level of autofluorescence in the gallstone-mimicking conditions (see above and Discussion). We therefore grew STy biofilms in cholesterol-coated tubes for two days and obtained direct visualizations of surface-attached communities by scanning electron microscopy. We observed dense aggregates of the wild type parent having a rich network of extracellular matrix that connected the cells, while the strains defective in *rpoS*, *sthC*, *waaZ* and *tviD* formed smaller STy aggregates with poor network connections (Fig. 4). Our high-resolution observations of cholesterol-attached STy communities by scanning electron microscopy consolidated our in vitro measurements of the sessile biomass formed by the STy strains by the standard crystal violet staining assay (Fig. 3, above). Overall, we demonstrated that the Tn-ClickSeq approach enabled the identification of previously unidentified players that drive the formation of atypical STy biofilms.

### RpoS activates transcription of STy biofilm matrix genes

**Figure 5:**
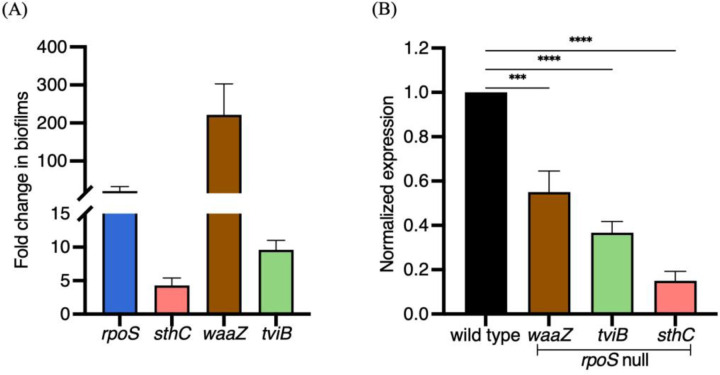
Steady-state levels of biofilm components are increased in gallstone-mimicking conditions and RpoS is required for the expression of biofilm matrix genes. Real time RT-qPCR analysis showed (A) a significant increase in transcription of *rpoS*, *sthC*, *waaZ* and *tviB* in the cholesterol-attached fraction compared to the planktonic fraction in the wild type H58 parent and (B) In the *rpoS* null strain, there was a significant decrease of biofilm matrix components *waaZ*, *tviD* and *sthC*. *rrsA* transcript levels were used as an internal control. N = 3, in triplicates, error bars represent Mean ± SD, *p ≤ 0.05, **p ≤ 0.01, ***p ≤ 0.001 and ****p ≤ 0.0001 by one-way ANOVA.

We next wanted to determine the possible role of RpoS in regulating the expression of STy biofilm components, including: SthC, WaaZ and the Vi capsule. We first investigated if the expression of *rpoS*, *sthC*, *waaZ* and *tviB* (the *tviBCDE* operon mediates biosynthesis of Vi-polysaccharide^[57]^), was up-regulated in biofilm-inducing conditions. We isolated planktonic and biofilm sub-populations from wild type strain grown in cholesterol-coated tubes and measured steady state transcript levels by reverse transcription quantitative real-time PCR (RT-qPCR). Indeed, we detected an increase in the transcription of *rpoS* (~20-fold), *sthC* (~4-fold), *waaZ* (~200-fold) and *tviB* (~10-fold) in biofilms formed by the H58 parent in gallstone-mimicking conditions (Fig. 5A).

In order to investigate the exact role of RpoS in favoring the biofilm lifestyle, we grew the wild type parent H58 and its *rpoS* null derivative in cholesterol-coated tubes in vitro and isolated total RNA from the biofilm fractions after two days. We then compared the transcription of matrix encoding genes *waaZ*, *sthC* and *tviB* by RT-qPCR and observed a positive effect of RpoS at each of these loci (Fig. 5B). Transcription of the matrix-encoding genes was clearly down-regulated when RpoS was absent (Fig. 5B). This result established a molecular link between the stress sigma factor, RpoS, and development of STy cholesterol-attached biofilms.

### RpoS regulates infection outcomes in zebrafish larvae and drives persistence in vivo

**Figure 6:**
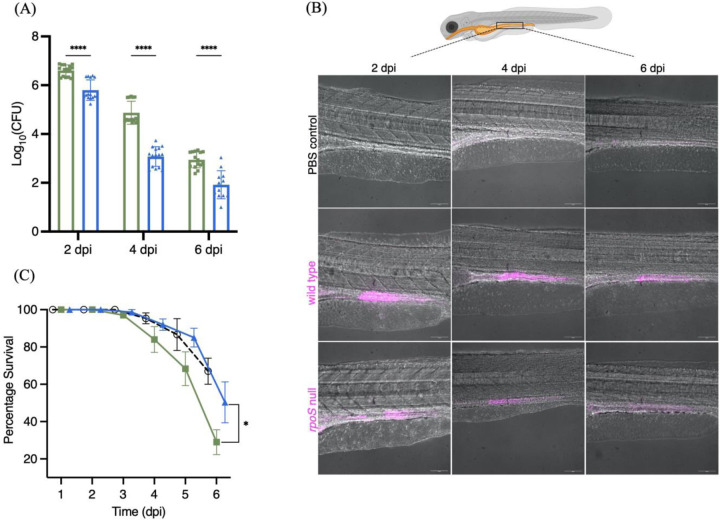
STy colonizes the intestines of chronically infected zebrafish, and RpoS is required for persistent colonization in vivo. (A) The number of STy colonies recovered from persistently infected whole larvae was drastically reduced in the *rpoS* null strain (blue bars) compared to the wild type (green bars) at days 2, 4 and 6 post infection. Control larvae exposed to PBS were void of *Salmonella*. N = 3, five infected larvae from each group, error bars represent Mean ± SD and ****p ≤ 0.0001 by two-way ANOVA with Sidak’s multiple comparison tests. (B) Representative merged images (red channel and bright field) of live zebrafish larvae showing the presence of wild type mCherry-expressing *S.* Typhi in the gut. There was a stark reduction of bacteria in the infections using the mCherry-tagged *rpoS* null strain at 2, 4 and 6 dpi. No fluorescence was detected for the PBS control at all time points. The cartoon on top depicts an infected zebrafish larva with the highlighted intestinal region was adapted from BioRender. 20x magnification, Scale bar = 10 µm. (C) Larvae infected with the *rpoS* null strain (blue line) survived longer and showed a significant increase in the percentage of survival at 6 dpi compared to the wild type H58 (green line). The survival rate of the PBS control is shown as a dotted black line. N = 3 with 30 to 60 larvae in each group, error bars represent Mean ± SD, *p ≤ 0.05 by two-way ANOVA with Sidak’s multiple comparison tests.

Since the formation of STy biofilms plays a crucial role in enabling persistence in human carriers, we investigated the role of RpoS in persistent infections in a zebrafish model. We replicated the natural mode of Typhi entry by employing static immersions of zebrafish larvae in tank water contaminated with specific doses of *S.* Typhi strains. We exposed 5 days post-fertilization (dpf) zebrafish larvae to 10^8^ cfu/ml of mCherry-expressing wild type and the isogenic *rpoS* null mutant in system water for 24 h. After 24 h, the larvae were shifted to plain system water and monitored for six days under standard conditions of zebrafish husbandry^[adapted from 29]^. Control larvae were exposed to an equal volume of PBS. We discovered that *S.* Typhi colonization of infected larvae was reduced when RpoS was absent, as evident from both bacterial load measurements (Fig. 6A), as well as real-time visualizations at 2, 4 and 6 dpi using confocal fluorescence microscopy (Fig. 6B). At longer times of 4 and 6 dpi, wild type *S.* Typhi clearly persisted in the intestine (Fig. 6A, B), as also observed previously in a mouse model of STm persistence^[58]^. Finally, persistence was strongly correlated with pathogenesis outcomes as larvae infected with the *rpoS* null strain survived in significantly greater numbers than the wild type infections at 6 dpi (Fig. 6C). In fact, the survival rate of *rpoS* infections was similar to the uninfected PBS control from 1 to 6 dpi, strongly emphasizing the requirement of RpoS for enabling STy survival in long term infections. Monitoring infected larvae beyond 6 dpi is challenging, owing to a greater requirement of live food (Paramecia) which masks pathogen-driven physiological effects^[59 and see Discussion]^. In conclusion, we revealed a positive role for RpoS in chronic STy infections and established that RpoS was necessary for persistence in vivo.

## Discussion

The dangerous transmission of *S.* Typhi from seemingly healthy but chronically infected individuals is well documented and the first cases in the United States of Mary Mallon, ‘Typhoid Mary’, and Mr. N in the United Kingdom, were reported in the early 1900s^[60, 61]^. Since then several epidemiological studies have established that persistent colonization of STy in the gall bladders of asymptomatic patients forms the basis for typhoid carriage^[reviewed in 18, 62]^. Despite clear evidence of the role of STy biofilms in spreading the disease, previous studies have failed to delineate any genetic mechanisms that regulate the development of gallstone biofilms in STy. This is chiefly because biofilms formed by *S.* Typhimurium have been employed as a surrogate for understanding the multicellular behavior of *S.* Typhi^[63–65]^. In the present work, we studied STy biofilms directly and discovered that the pathways for biofilm formation in STy and STm were completely different. More importantly CsgD, the master regulator of STm surface-attached communities, was entirely dispensable for the formation of STy sessile aggregates on cholesterol-coated surfaces. As a result, matrix production in STy biofilms did not require curli fibers or biosynthesis of O-Antigen via the *yihO/P* system.

We grew a transposon library in the STy parent strain H58 as biofilms in vitro in gallstone-mimicking conditions and developed Tn-ClickSeq analysis to map the transposon genome junctions enriched in the planktonic and biofilm sub-populations. We found Tn-genome junctions in 47% and 20% of total sequencing reads from the planktonic and biofilm fractions, respectively. These differences indicated that presumably inactivating transposon insertions were tolerated in only a low number of genes in biofilms, which correlated with their enrichment in the planktonic fraction. Also the technical challenges of isolating genomic DNA with high efficiency from STy cells attached to cholesterol-coated surfaces may affect the total sequencing yields, leading to very low insertion indices in biofilms. Nevertheless, our Tn-ClickSeq analysis generated novel insights by identifying Sth fimbriae, Vi capsule and the lipopolysaccharide core as structural components and IraP as a regulator of STy cholesterol-attached biofilms. Our comprehensive analysis of the STy biofilms also opens up exciting new directions for mapping the complete genetic signature of STy carriage.

The discovery of IraP is significant because it motivated our subsequent investigations on the significance of the downstream starvation-induced sigma factor, RpoS, during STy biofilm development. We established that the formation of STy biofilms required RpoS and determined its role in activating the transcription of the extracellular matrix components: Sth fimbriae, Vi capsule and lipopolysaccharide. Interestingly, the binding sites for RpoS have been identified in the *waaZ* promoter in *E. coli* ^[66]^. A previous study had also described a regulatory interplay between RpoS and the TCRS RcsC/B to regulate Vi capsule synthesis under different osmotic conditions^[67]^.

A high degree of autofluorescence and the non-specific binding of fluorescent dyes and antibodies on cholesterol surfaces prevents the visualization of mature STy biofilms using high-resolution fluorescence microscopy techniques. Although some protocols have been developed to improve the fluorescence imaging of STy aggregates in vitro and ex vivo, crucial controls that included STy strains defective in forming biofilms were absent^[68–70]^. Therefore, in order to clearly visualize STy biofilms attached to cholesterol surfaces, we employed scanning electron microscopy and observed distinct aggregates of the wild type H58 parent and substantially reduced aggregate formation in the STy biofilm mutants validated from our Tn-ClickSeq analysis.

Our use of zebrafish larvae as a heterologous host for *S.* Typhi validated the important role of RpoS in enabling *S.* Typhi colonization in long-term infections. Reducing STy persistence by inactivating *rpoS* significantly decreased the pathogen load and prolonged host survival. Following STy infections beyond 6 dpi may be possible by rearing germ-free zebrafish larvae using established methods^[71]^.

Finally, a strong correlation has been observed between the ability to form biofilms and the duration of STy shedding and carriage in typhoid patients from Pakistan^[72]^. Most recent phylogenomic analysis has proposed that the ancestral H58 haplotype originated in a chronic carrier from India and evolved to give rise to the three sub-lineages that cause a majority of typhoid infections in Asia and Africa^[23]^. In the light of these results, it is tempting to propose that RpoS contributes to niche adaptation in *S.* Typhi by activating the formation of biofilms in chronic carriers.

## Methods

### Bacterial strains and growth

The bacterial strains and plasmids used in this study are listed in Supplementary Table 1. *S.* Typhi strains were routinely grown in Luria-Bertani broth (LB) or Nutrient broth (NB) (BD Difco) medium with shaking at 275 rpm at 37°C in the presence of 100 μg/ml Ampicillin, 12.5 μg/ml Tetracycline, 25 μg/ml Chloramphenicol or 50 μg/ml Kanamycin (Millipore Sigma) when necessary. For the growth of STy cholesterol-attached biofilms, a modified NB medium (NB*) containing 3% w/v NB, 1.75% w/v sodium chloride, 0.25% w/v potassium chloride, 1% w/v sodium choleate and 2% w/v glucose (Millipore Sigma) was used ^[36]^. For observing the rdar morphotype, 40 μl of overnight LB broth cultures of STm wild type strain 14028s and STy wild type strain H58 were spotted on agar plates containing 1% w/v Tryptone and 0.5% w/v Yeast Extract supplemented with Congo Red (40 μg/ml) (Millipore Sigma) and kept at 30°C for two days.

### Molecular Biology techniques

All DNA manipulation procedures were carried out according to^[73]^ using reagents procured from Qiagen, Millipore Sigma or Invitrogen. All transformations in STy wild type strain H58 were performed by standard electroporation protocols^[73]^. Polymerase chain reactions (PCR) were carried out using oligonucleotides as listed in the Supplementary Table 2 following standard protocols^[73]^.

### Strain construction

The *ssrB* null mutation in H58 strain was generated by transducing the *ssrB::kan* allele from the STm strain DW85 using standard P22 transduction protocols^[74]^. Other gene deletions in the STy wild type strain H58, as listed in Supplementary Table 1, were generated by the lambda *red* homologous recombination technique as described in^[75]^. Briefly, plasmids pKD3, pKD4 or the *TetRA* DNA were used to generate linear DNA fragments by PCR using gene-specific hybrid primers as listed in Supplementary Table 2. A 10 ml LB broth culture of H58 transformed with the plasmid pKD46 (containing 100 μg/ml Ampicillin and 20 mM Arabinose) was used to electroporate 600 ng to 1 μg of purified PCR product following the protocol as described in^[75]^. The cells were recovered for at least 6 h at 275 rpm/30 °C after which the cells were harvested and plated on respective selective plates. Chromosomal deletions were confirmed by PCR using flanking primer pairs as listed in Supplementary Table 2.

### STy cholesterol-attached biofilms in gallstone-mimicking conditions

STy biofilms were routinely grown following protocols adapted from^[36, 38]^. Sterile 1.5 ml Eppendorf tubes containing 200 μl of 10 mg/ml cholesterol (Millipore Sigma) in diethyl ether were air dried aseptically (2–3 h) and 20 μl of STy strains grown overnight in LB/NB was added to 180 μl NB* medium. The tubes were incubated for two days at 275 rpm at 37 °C. Cholesterol-coated tubes containing only 200 μl NB* medium served as controls in all the experiments. For monitoring the time course of biofilm formation, the cholesterol-coated tubes were further incubated for four and six days, with fresh 200 μl NB* medium replaced every two days.

### Crystal violet staining assay

The amount of STy cholesterol-attached biofilms in gallstone-mimicking conditions was estimated using crystal violet staining assays adapted from^[10, 38]^. The supernatant/growth medium was removed, and each tube was washed once with 400 μl of Phosphate-buffered Saline (PBS). The attached biofilms were then stained with 200 μl of 0.1 % w/v crystal violet solution (filtered using Whatman Grade 1 filter paper) for five minutes at room temperature (RT). This was followed by washing once with 400 μl PBS and addition of 200 μl absolute ethanol. Appropriate dilutions were then measured for absorbance at 595 nm using an iMark^™^ Microplate Absorbance Reader. Each experiment was performed in triplicates or pentuplicates.

### Isolation of fractions for Tn-ClickSeq libraries

A *TnTMDH5deloriR6K* genome library in the *S.* Typhi wild type strain H58 (H58-Tn) was kindly provided by Dr. Stephen Baker, University of Cambridge, UK. Details on the construction of the library have been previously described in^[46]^. A 1 μl loop of frozen stock of the H58-Tn library was inoculated in 10 ml NB* cultures and incubated overnight at 275 rpm at 37 °C. This was the ‘input’ fraction, which was used to prepare thirty tubes of cholesterol-attached biofilms in gallstone-mimicking conditions. After two days, the culture supernatants were removed and pooled to obtain the ‘planktonic’ fraction. To isolate the ‘biofilm’ fraction, 400 μl of PBS was added to each tube followed by sonication in a XUB Digital Ultrasonic Bath (Grant Instruments) for 20 minutes, maximum power, no leap, at RT. The harvested biomass was pooled, and the sonication step was repeated thrice. The final pool at the end of four sonication cycles was the ‘biofilm’ fraction.

### Genomic DNA isolation for generating Tn-ClickSeq libraries

Planktonic and biofilm fractions were centrifuged at 24,000 g at 4 °C for 1.5 h in a Beckman Coulter Avanti J-26XP centrifuge. Respective supernatants were discarded, and the pellets were stored on ice. 1 ml of the input fraction was also centrifuged at 15,500 g at 4 °C for 10 min and the pellet was stored on ice. Each of the pellet fractions were then resuspended in 600 μl TE buffer (pH 8.0) with 40 μl 10% w/v sodium dodecyl sulphate, 4 μl Proteinase-K and 2 μl RNaseE and mixed well by vortexing. Samples were incubated at 37 °C for 1 h, after which an equal volume of Phenol:Chloroform mixture (pH 6.7/8.0) was added, mixed and centrifuged at 15,500 g at 4 °C for 15 min. The upper aqueous phase was added to a fresh tube and an equal volume of chloroform was added, mixed, and centrifuged at 13,000 rpm at 4 °C for 15 min. The supernatants were removed, 2.5 to 3 volumes cold absolute ethanol was added, and stored overnight at −20 °C. DNA pellets were obtained by centrifuging the samples at 15,500 g at 4 °C for 15 min, followed by a 70% ethanol wash. The pellets were air dried, and DNA was resuspended in 40 μl of nuclease-free water.

### Preparation of Tn-ClickSeq libraries

For Tn-ClickSeq, genomic DNA from the input, planktonic and biofilm fractions was reverse transcribed using a reverse transcriptase (SSIII, Invitrogen) and Azido-NTPs. A Tn-specific reverse primer (3’21–39, Supplementary Table 2) was designed to the 3’ proximal end, 21 to 39 bp of transposon, with an overhang of the reverse complementary sequence of Illumina adapter (Supplementary Figure 4, Supplementary Table 2). 500 ng DNA was mixed with 1 µL of 5 µM primer, and 1 µL of 10 mM AzNTP/dTNP mixture (AzNTP:dNTP = 1:35). This initial reaction mix was heated at 95 °C for 5 min and then cooled to 50 °C in gradual steps of 0.1 °C per second. Other reaction components for the reverse transcription reaction were then added: buffer, DTT, SSIII (as per manufacturer’s protocol), and kept in a thermocycler at 50 °C for 50 min, after which the reaction was terminated at 95 °C for 5 min. This was followed by the standard ClickSeq protocol as previously described^[44, 76, 77]^. Briefly, immediately after denaturing, the DNA products were purified with Solid Phase Reversible Immobilization (SPRI) beads and click-ligated with a 5’-alkyne-modified adapter including a 12-nucleotide unique molecular identifier (UMI). The click-ligated product was then purified, barcoded, amplified with 18 to 20 cycles of PCR, and analyzed by agarose gel electrophoresis. The final Tn-ClickSeq libraries were subjected to pair-end sequencing on a NexSeq 550 platform at the Genomics core, UTMB.

### Bioinformatics analysis

We designed a bioinformatics pipeline to process the hybrid reads that originate from the inserted transposon and extend into the host genome (Supplementary Figure 5). The raw paired-end FASTQ reads were first pre-processed to trim the Illumina adapter, filter low-quality reads and extract UMIs using *fastp*^[78]^: -a AGATCGGAAGAGC -U --umi_loc read1 --umi_len 14 --umi_prefix umi -l 30. We then used *FASTX toolkit* (http://hannonlab.cshl.edu/fastx toolkit/index.html) to reverse complement the R2 reads for ease of downstream analyses. We filtered the reads that contained the 19 bp primer sequence (targeting the Tn) with 1 nucleotide mismatch allowance with *cutadapt*^[79]^: -a cctatagtgagtcgtatta -e 0.1 -O 19 -m 30 -- discard-untrimmed. To obtain the reads containing primer sequences, we further filtered reads that contained the last 10 nucleotides of the 3’ invert repeat (IR) with 0% error rate allowance with *cutadapt*: - a ctgtctctta −e 0 −O 10 −m 30 --discard-untrimmed. After trimming the IR sequence, the rest of IR-containing reads were mapped to the H58 genome (https://www.ncbi.nlm.nih.gov/datasets/genome/GCF_001051385.1/) with *hisat2*^[80]^, and then processed with *SAMtools*^[81]^: view/sort/index. We de-duplexed the data to minimize PCR bias with umi_tools^[82]^: dedup --method=unique. Finally, the locations of the transposon insertion sites were extracted with *BEDTools*[83]: genomeCoverageBed: −3 −bg. Raw sequencing data is available at the Sequence Read Archive, Project ID, PRJNA1029173.

### Gene mapping and target analysis

The number of insertion reads at each insertion site revealed by Tn-ClickSeq were ratiometrically normalized across different samples. This was followed by the assignment of gene names and annotations within H58 (GCF_001051385.1). We normalized the number of insertions to the length of each annotated gene (per 1 Kbp length of gene) to obtain the insertion indices for each samples. Matrices of all the normalized insertion dataset were then processed with DESeq2 to identify genes enriched or depleted in each conditions and perform Principal component analysis^[84]^. Hierarchical clustering was conducted with Cluster 3.0 (http://bonsai.hgc.jp/~mdehoon/software/cluster/), followed by TreeView (http://jtreeview.sourceforge.net/) to build the graphic map. Gene ontology analysis was conducted with GeneOntology web server (http://geneontology.org/) with *Salmonella* Typhimurium as a reference.

### RNA isolation

Planktonic and biofilm fractions were centrifuged at 24,000 g at 4 °C for 1.5 h in a Beckman Coulter Avanti J-26XP centrifuge. Respective supernatants were discarded, and the pellets were either stored at −80 °C, or immediately processed for total RNA isolation by the Trizol method^[85]^. Briefly, the pellets were resuspended in 1 ml TRIzol reagent (Life Technologies) and incubated at RT for 5 min. 200 μl chloroform was added, mixed well, and incubated for 3 min at RT. The mixtures were centrifuged at 15,500 g for 15 min in cold and 500 μl isopropanol was added to the supernatants. The samples were transferred to −20 °C overnight, after which the RNA was pelleted by centrifugation at 15,500 g for 15 min in cold. This was followed by a 75% ethanol wash. The pellets were air dried, and the RNA was resuspended in 20 μl of nuclease-free water.

### RT-qPCR

1 μg of total RNA extracted from the biofilm fraction of two days old cholesterol-attached biofilms was used for a reverse transcription reaction with iScript Supermix (Bio-Rad) according to the manufacturer’s protocol. This was followed by amplifying 50 ng cDNA by real-time qPCR (RT-qPCR) using SsoFast EvaGreen Supermix (Bio-Rad) and internal primers specific for *rpoS*, *sthC*, *waaZ* and *tviB*; *rrsA* was used as a normalization control (Supplementary Table 2). The annealing temperature for all the primer pairs was 56 °C. All experiments were performed in triplicates with at least three independently isolated RNA preparations. Relative expression was determined using the 2^−ΔΔC^_T_ (Livak) method as described in^[85]^ and plotted using the GraphPad Prism 10 software.

### Scanning electron microscopy

STy cholesterol-attached biofilms were grown in gallstone-mimicking conditions for two days and after removal of the growth medium were fixed in a primary fixative containing 2.5% w/v formaldehyde (made from paraformaldehyde), 0.1% v/v glutaraldehyde, 0.01% v/v trinitrophenol and 0.03% w/v CaCl_2_ in 0.05M sodium cacodylate buffer (pH 7.3). Samples were post-fixed in 1% w/v OsO_4_ in cacodylate buffer, dehydrated in ethanol and infiltrated with hexamethyldisilazane (HMDS) to prevent cracking during drying. After air drying, the conical parts of the Eppendorf tubes were cut into strips, mounted on SEM specimen holders (metal stubs) and sputter-coated with iridium in an Emitech K575X (Emitech, Houston, TX) sputter coater for 30 seconds at 20 mA, at the Electron Microscopy Laboratory, Department of Pathology, UTMB. The samples were examined in a JEOL JSM-6330F Scanning electron microscope at the Texas Center for Superconductivity, University of Houston, at 4 μA and 2 kV.

### Zebrafish husbandry

All the protocols used for zebrafish experiments were approved by the University of Texas Medical Branch Institutional Care and Use Committee. Adult and larval zebrafish were maintained using standard husbandry procedures^[86]^ at our in-house satellite facility. The wild type AB line was used for lifespan analysis and Typhi load measurements and the *casper* line was used for visualizing mCherry-tagged STy strains in infected larvae by confocal fluorescence imaging. Eggs were obtained by the natural spawning method^[86]^ and kept at 28 °C in embryo medium containing methylene blue for two days. At 2 days post-fertilization (dpf), the larvae were shifted to an embryo medium containing 25 μg/ml gentamicin for 6 h, after which they were transferred to a sterile embryo medium at 28 °C. Fresh embryo medium was replaced daily.

### Static immersions of zebrafish larvae

All infections were performed using mCherry-tagged STy strains, as listed in Supplementary table 2. For static immersions, 1 ml of an overnight LB broth culture of mCherry-tagged STy strains was inoculated in 10 ml LB broth containing 100 μg/ml ampicillin and grown for 4.5 hours at 275 rpm at 37 °C. Growth was normalized by measuring absorbance at 600 nm. The strains were harvested by centrifugation at 4,200 g for 15 min at RT and resuspended in 500 μl sterile embryo medium. Ten larvae 5 dpf were added to 8 ml embryo medium in a 6-well polystyrene plate, followed by the addition of 80 μl of harvested STy cultures (10^9^ cfu/ml). Equal infection doses across strains were verified using aliquots of the harvested STy cultures by the total viable counting method. Larvae exposed to equal volume of PBS served as controls. After 24 h, the larvae were transferred to fresh embryo medium in a 6-well plate. Fresh embryo medium was replaced daily. The larvae were fed once daily from 9 dpf, as recommended^[59, 86]^. Typically, infections were performed with thirty to sixty larvae in each group.

### Bacterial load estimates from infected larvae

For enumerating the STy load at 2, 4 and 6 dpi, infected larvae were isolated and added to embryo medium containing 60 to 100 μg/ml buffered Tricaine. The anesthetized larva was transferred to a 1.5 ml Eppendorf tube containing 200 μl PBS and homogenized well with a motorized micropestle. The homogenate was serially diluted and colony forming units (cfu) were estimated by the total viable counting method on LB agar plates containing 100 μg/ml ampicillin. For each time point, five larvae were used from each group. PBS control larvae remained sterile. The experiment was repeated at least three times.

### Lifespan analysis

Infected larvae were checked daily under a dissection microscope, and the percentage survival was scored. Larvae that did not show any heart beats were considered dead.

### Confocal fluorescence imaging

Five larvae from each group were withdrawn at 2, 4 and 6 dpi and anesthetized in an embryo medium containing 60 to 100 μg/ml buffered Tricaine. These were then mounted on 35 mm glass-bottomed dishes with a drop of 0.8% low melting point agarose (supplemented with 60 to 100 μg/ml buffered tricaine). Images were acquired on an Olympus SpinSR-10 Yokogawa spinning disk confocal microscope fitted with an ORCA Fusion sCMOS camera (Hamamatsu) using a 20x objective (NA 0.8, Olympus), 561 CSU and a step size of 1.5 μ in the Z-dimension. Images were analyzed using Image J software.

### Statistics

The Prism 10 software was used to plot all graphs and perform statistical analyses.

## Supplementary Material

Supplement 1

## Abbreviations:

rdar: red, dry and rough
cfu: colony forming unit
RT: room temperature
h: hour
PCR: Polymerase chain reaction
RT-qPCR: Reverse transcription quantitative real-time PCR
Tn: Transposon
NGS: next-generation sequencing
TCRS: two-component regulatory system
