# Supplementary material for: RpoS activates formation of Salmonella Typhi biofilms and drives persistence in the gall bladder
Source: bioRxiv. 2024 Oct 7:2023.10.26.564249. Originally published 2023 Oct 26. Preprint. [Version 3] doi: 10.1101/2023.10.26.564249 (PMC10634867; doi:10.1101/2023.10.26.564249)
Supplement: Supplement 1 [file NIHPP2023.10.26.564249v3-supplement-1.pdf]

bioRxiv preprint doi: https://doi.org/10.1101/2023.10.26.564249; this version posted October 7, 2024. The copyright holder for this preprint (which was not certified by peer review) is the author/funder, who has granted bioRxiv a license to display the preprint in perpetuity. It is made available under aCC-BY 4.0 International license.

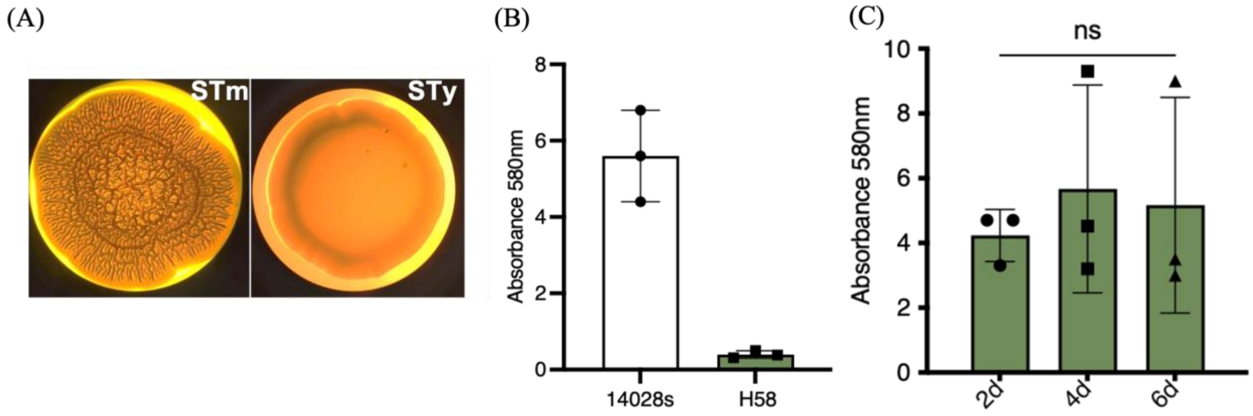

Supplementary Figure 1: H58 forms 'atypical' biofilms. (A) Examination of a macrocolony of *S.* Typhimurium (STm) displaying a red, dry and rough (rdar) morphotype on Luria-Bertani (LB) agar without salt medium containing the congo red dye, while *S.* Typhi (STy) are 'smooth' and lack the rdar morphology. (B) Wild type STy strain H58 was unable to form biofilms compared to the wild type STm strain 14028s, when grown in LB broth without salt medium in polystyrene plates at two days as determined by a crystal violet staining assay and (C) The cholesterol-attached biomass formed by wild type H58 did not increase significantly at days 4 and 6 compared to day 2, as determined by a crystal violet staining assay. Growth medium added to cholesterol-coated Eppendorf tubes was used as the control and subtracted from all measurements. N = 3, in at least triplicates, error bars represent Mean ± SD, ns = not significant by one-way ANOVA.

bioRxiv preprint doi: https://doi.org/10.1101/2023.10.26.564249; this version posted October 7, 2024. The copyright holder for this preprint (which was not certified by peer review) is the author/funder, who has granted bioRxiv a license to display the preprint in perpetuity. It is made available under aCC-BY 4.0 International license.

(A)

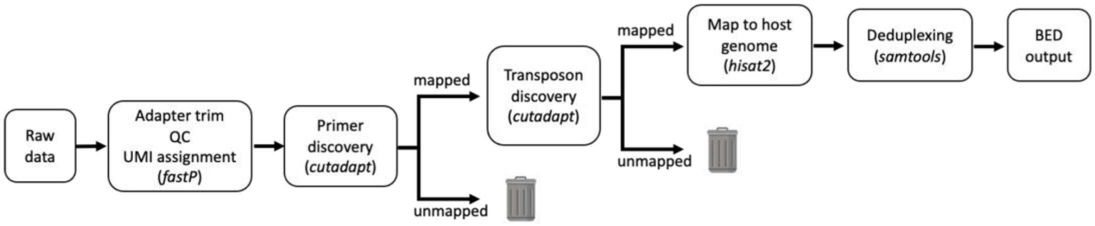

(B)

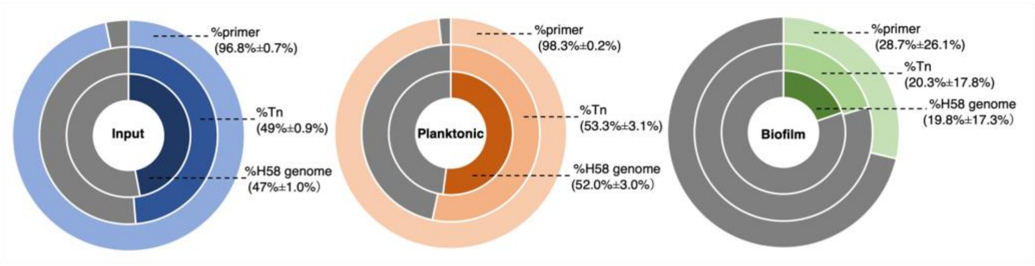

(C)

Sample	Raw reads (after QC)	Reads with primer	Reads with Tn	Reads mapping to H58 genome
Input#1	14, 256, 570	13, 914, 412	7, 099, 879	6, 891, 853
Input#2	11, 418, 976	10, 962, 217	5, 572, 442	5, 365, 704
Input#3	8, 352, 374	8, 093, 450	3, 974, 593	3, 825, 148
Planktonic#1	15, 228, 533	15, 000, 105	8, 234, 051	8, 013, 378
Planktonic#2	13, 131, 165	12, 881, 673	7, 440, 776	7, 260, 709
Planktonic#3	16, 164, 559	15, 905, 926	7, 942, 465	7, 759, 788
Biofilms#1	406, 289	23, 158	20, 738	20, 543
Biofilms#2	540, 650	82, 179	57, 380	55, 349
Biofilms#3	459, 357	299, 041	207, 996	202, 089
Negative control	18, 888, 763	18, 359, 878	2, 433	2, 331

(D)

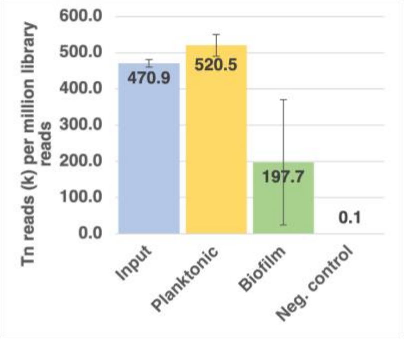

bioRxiv preprint doi: https://doi.org/10.1101/2023.10.26.564249; this version posted October 7, 2024. The copyright holder for this preprint (which was not certified by peer review) is the author/funder, who has granted bioRxiv a license to display the preprint in perpetuity. It is made available under aCC-BY 4.0 International license.

Supplementary Figure 2: (A) A flowchart of the bioinformatics pipeline employed for Tn-ClickSeq analysis depicting the filtering of raw reads containing both primer and partial Tn sequences, the subsequent identification of genome insertion sites using the reference H58 genome and lastly de-duplexing to remove PCR biases. (B) Percentages of reads that matched the primer, transposon sequence (IR sequence) and the host genome from left to right: input, planktonic and biofilm sub-populations. (C) Sequencing yields of the respective input, planktonic and biofilm Tn-ClickSeq libraries in triplicates and of an H58 strain without any transposon insertions as a negative control and (D) Graphical representation of Tn insertion sites per one million of raw reads showing an average of 470,000 reads for the input fraction, 520,000 for the planktonic fraction and 197,000 for the biofilm fraction. A similar analysis using an H58 strain without any transposon insertions resulted in only 100 false positives.

bioRxiv preprint doi: https://doi.org/10.1101/2023.10.26.564249; this version posted October 7, 2024. The copyright holder for this preprint (which was not certified by peer review) is the author/funder, who has granted bioRxiv a license to display the preprint in perpetuity. It is made available under aCC-BY 4.0 International license.

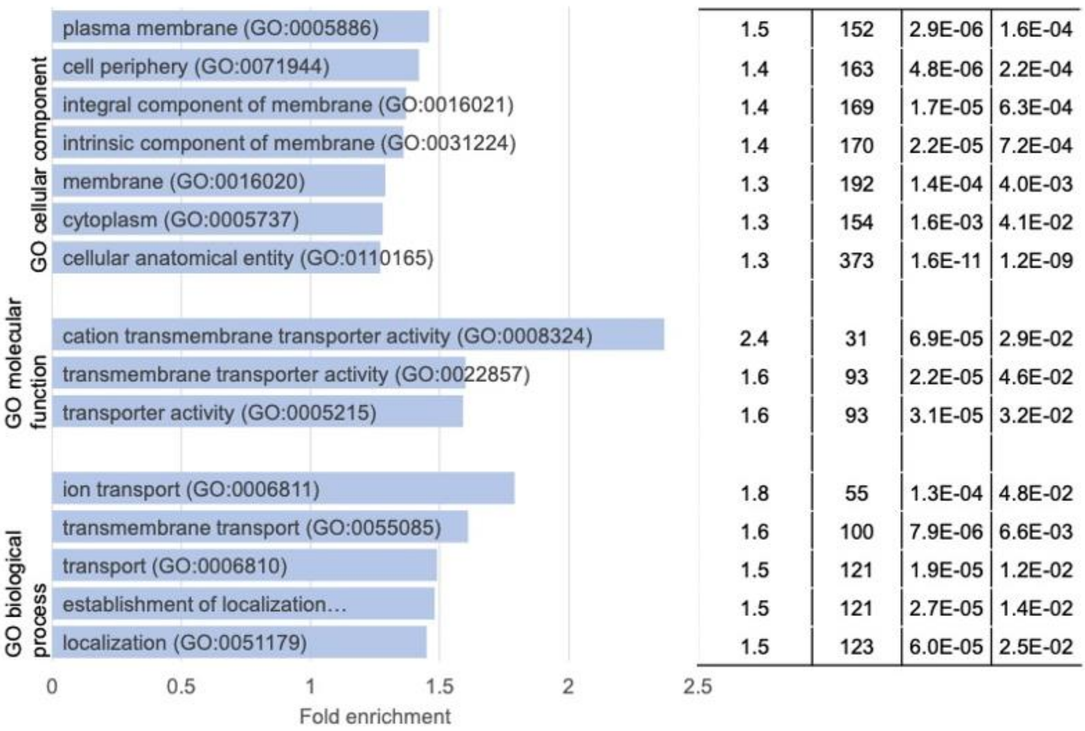

Fold enrichment	# Genes	P value	FDR
1.5	152	2.9E-06	1.6E-04
1.4	163	4.8E-06	2.2E-04
1.4	169	1.7E-05	6.3E-04
1.4	170	2.2E-05	7.2E-04
1.3	192	1.4E-04	4.0E-03
1.3	154	1.6E-03	4.1E-02
1.3	373	1.6E-11	1.2E-09
2.4	31	6.9E-05	2.9E-02
1.6	93	2.2E-05	4.6E-02
1.6	93	3.1E-05	3.2E-02
1.8	55	1.3E-04	4.8E-02
1.6	100	7.9E-06	6.6E-03
1.5	121	1.9E-05	1.2E-02
1.5	121	2.7E-05	1.4E-02
1.5	123	6.0E-05	2.5E-02

Supplementary Figure 3: A Gene Ontology analysis of transposon insertion sites in 1515 genes that were enriched in the planktonic sub-population as compared to biofilms showed a significant enrichment of cell membrane components, transmembrane ion transport pathways and other membrane related activities.

bioRxiv preprint doi: https://doi.org/10.1101/2023.10.26.564249; this version posted October 7, 2024. The copyright holder for this preprint (which was not certified by peer review) is the author/funder, who has granted bioRxiv a license to display the preprint in perpetuity. It is made available under aCC-BY 4.0 International license.

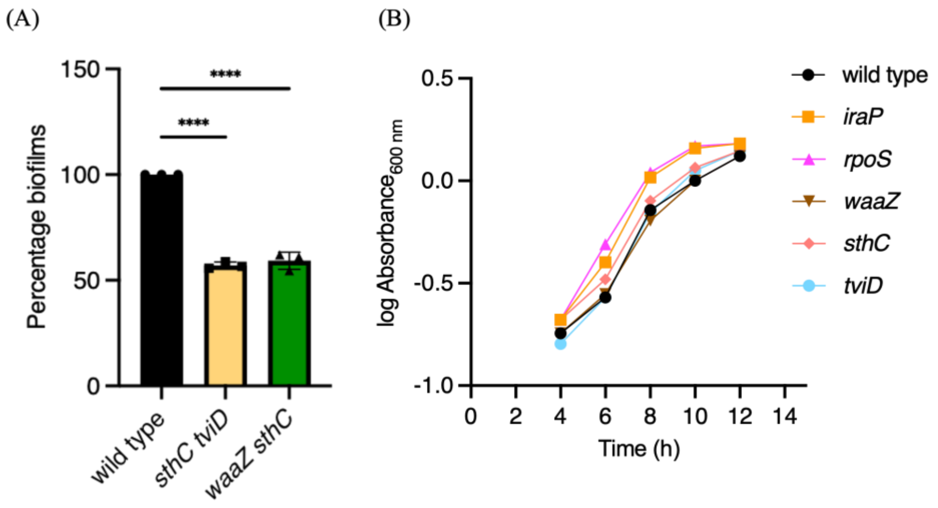

Supplementary Figure 4: (A) Double null mutant derivatives of an H58 parent defective for Sth fimbriae and Vi-polysaccharide (*sthC tviD*), and for LPS biosynthesis and Sth fimbriae (*waaZ sthC*) showed a reduced ability to form cholesterol-attached biofilms, by around 50%, compared to the wild type parent. N = 3, in at least triplicates, error bars represent Mean ± SD, in a crystal violet staining assay. Growth medium added to cholesterol-coated Eppendorf tubes was used as the control and subtracted from all measurements, ****p ≤ 0.0001 by one-way ANOVA and (B) Biofilm mutants are not compromised in planktonic growth - Strains deleted of *iraP*, *rpoS*, *waaZ*, *sthC* or *tviD* and the wild type H58 strain were grown for 12 hours in Luria-Bertani broth at 37°C/250 rpm and the absorbance at 600 nm was measured every 2 hours.

bioRxiv preprint doi: https://doi.org/10.1101/2023.10.26.564249; this version posted October 7, 2024. The copyright holder for this preprint (which was not certified by peer review) is the author/funder, who has granted bioRxiv a license to display the preprint in perpetuity. It is made available under aCC-BY 4.0 International license.

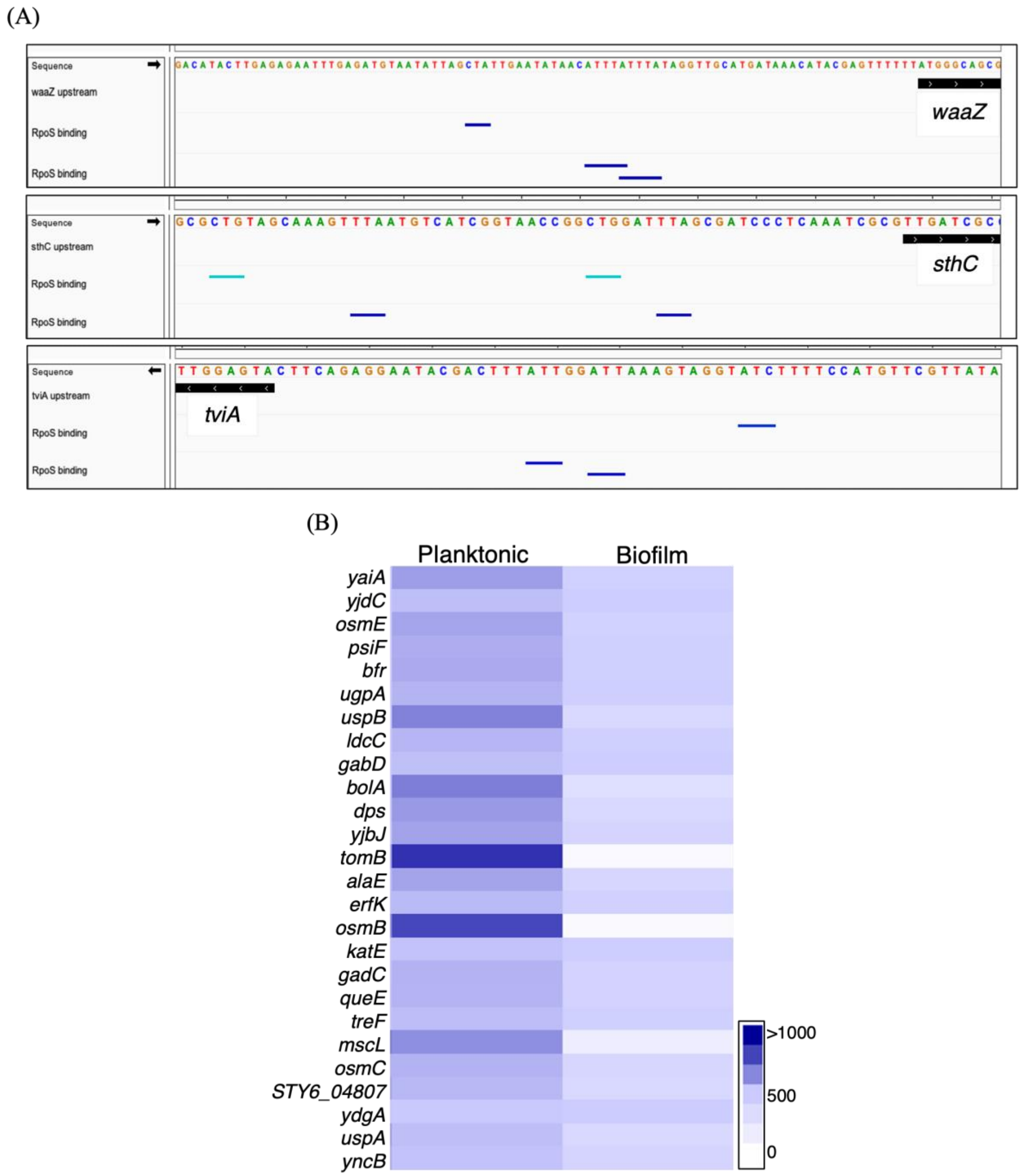

bioRxiv preprint doi: https://doi.org/10.1101/2023.10.26.564249; this version posted October 7, 2024. The copyright holder for this preprint (which was not certified by peer review) is the author/funder, who has granted bioRxiv a license to display the preprint in perpetuity. It is made available under aCC-BY 4.0 International license.

Supplementary Figure 5: (A) Sequences of upstream regulatory regions of *waaZ*, *sthC* and *tviA* are shown as snapshots from the IGV genome browser with the RpoS binding sites, an extended -10 element followed by an AT-rich sequence, highlighted as dark blue lines (total conservation) and cyan lines (partial conservation) and (B) Hierarchical clustering of a subset of Tn-ClickSeq targets showing an enrichment of RpoS-regulated genes[59,60] in the planktonic library. For example, these include genes that confer acid resistance (*gadC*), adaptation to osmotic (*osmB*) and other environmental stresses (*psiF*, *uspA*, *uspB*), encode stress responsive regulators (*bolA* and *dps*), an iron storage protein (*bfr*), a toxin (*tomB*) and a mechanosensitive channel (*mscL*).

bioRxiv preprint doi: https://doi.org/10.1101/2023.10.26.564249; this version posted October 7, 2024. The copyright holder for this preprint (which was not certified by peer review) is the author/funder, who has granted bioRxiv a license to display the preprint in perpetuity. It is made available under aCC-BY 4.0 International license.

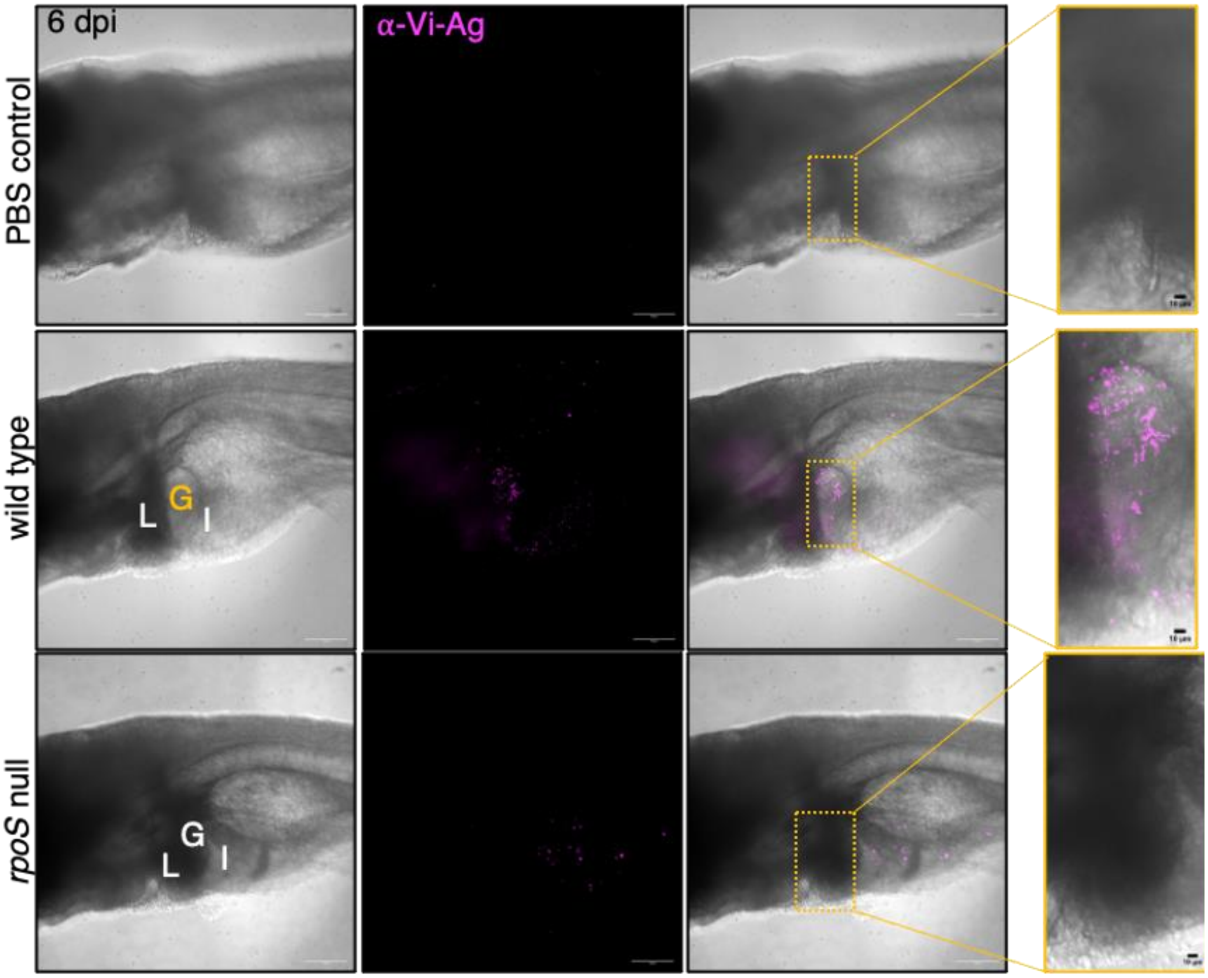

bioRxiv preprint doi: https://doi.org/10.1101/2023.10.26.564249; this version posted October 7, 2024. The copyright holder for this preprint (which was not certified by peer review) is the author/funder, who has granted bioRxiv a license to display the preprint in perpetuity. It is made available under aCC-BY 4.0 International license.

Supplementary Figure 6: Representative whole-mount immunohistochemistry images showing the successful detection of anti-Vi-polysaccharide antibody signal (magenta) in the gall bladder region, as marked by a yellow rectangle in the merged image of a wild type infected larva at 6 dpi (middle right panel). Liver (L) and intestine (I) are marked in the bright field image on the left to pinpoint the gall bladder (G) position. The presence of Vi-antigen-positive STy clusters in the gall bladder is highlighted in the respective zoomed-in image (middle right). The gall bladder region, as marked by a yellow rectangle, of an *rpoS* null infected larva remained negative for anti-Vi-antigen antibody staining (lower panel, zoomed-in image on the right). No fluorescence was detected in the uninfected PBS control (top panel). 20X magnification, Scale bar = 100 μm for all images except for zoomed-in images with 10 μm scale bars. N = 3 with 5 to 10 larvae analyzed in each group.

bioRxiv preprint doi: https://doi.org/10.1101/2023.10.26.564249; this version posted October 7, 2024. The copyright holder for this preprint (which was not certified by peer review) is the author/funder, who has granted bioRxiv a license to display the preprint in perpetuity. It is made available under aCC-BY 4.0 International license.

Supplementary Table 1: List of bacterial strains and plasmids

Strain or plasmid	Description	Source or Reference
14028s	*Salmonella enterica* serovar Typhimurium	Lab collection
H58 ('wild type' in this study)	*Salmonella enterica* serovar Typhi	Stephen Baker, Cambridge University, UK
Ty2-b	*Salmonella enterica* serovar Typhi strain SGSC 2408	Salmonella Genetic Stock Center, University of Calgary, CA
CT18	*Salmonella enterica* serovar Typhi strain SGSC 4072	Salmonella Genetic Stock Center, University of Calgary, CA
CT117	*Salmonella enterica* serovar Typhi	Stephen Baker, Cambridge University, UK
ssrB	H58 (*ssrB*::*kan*)	This study
csgD	H58 (*csgD*::*cat*)	This study
csgA	H58 (*csgA*::*kan*)	This study
yihO	H58 (*yihO*::*kan*)	This study
yihP	H58 (*yihP*::*kan*)	This study
yihO yihP	H58 (Δ*yihO yihP*::*kan*)	This study
iraP	H58 (*iraP*::*kan*)	This study
rpoS	H58 (*rpoS*::*kan*)	This study
sthC	H58 (*sthC*::*kan*)	This study
waaZ	H58 (*waaZ*::*kan*)	This study
tviA	H58 (*tviA*::*cat*)	This study
tviB	H58 (*tviD*::*kan*)	This study
sthC tviD	H58 Δ*sthC* (*tviD::kan*)	This study
waaZ sthC	H58 Δ*waaZ* (*sthC::cat*)	This study
rpoSc	H58 (*rpoS*::*kan*, pBR322::*rpoS*)	This study
pUHE21-lacI^q::*rpoS*	pUHE21-lacI^q construct expressing His_6-RpoS from 14028s	Kim et al., 2021[62]
pBR322::*rpoS* plasmid	*rpoS* cloned between EcoRI and ScaI sites in pBR322	Roy Curtiss III, University of Florida, USA
pFPV::mCherry plasmid	mCherry cloned between XbaI and SphI sites in pFPV	Olivia Steele-Mortimer (Addgene plasmid # 20956 http://n2t.net/addgene:20956; RRID:Addgene_20956

Supplementary Table 2: List of oligonucleotides

Primers	Sequence
**Tn-ClickSeq**	
3'21-39	GTGACTGGAGTTCAGACGTGTGCTCTTCCGATCTTAATACGACTCACTATAGG [Black = Illumina p7 adapter, red = Tn-specific]
**Gene deletions**	

bioRxiv preprint doi: https://doi.org/10.1101/2023.10.26.564249; this version posted October 7, 2024. The copyright holder for this preprint (which was not certified by peer review) is the author/funder, who has granted bioRxiv a license to display the preprint in perpetuity. It is made available under aCC-BY 4.0 International license.

csgD knockout forward	GCAGCTGTCAGATGTGCGATTAAAAAAAGTGGAGTTTCATCATGTTTAATGT GTAGGCTGTAGCTGCTTC
csgD knockout reverse	CTCTGCTGCTACAATCCAGGTCAGATAGCGTTTCATGGCCTTACCGCCTGCCT CCTTAGTTCCTATTCCG
csgA knockout forward	CACCCAACGCTAATACCGTTACGACTTTTAAATCAATCAATCCGGTGTAGGC TGGAGCTGCTTC
csgA knockout reverse	AGGGCTTATGCCCTGTTTTTTATTAGCGCAGACGCTAAACATATGAATATCC TCCTTAG
yihO knockout forward	ATGTCTAATCATGATCCGCTAACGCTAAAGTTGAGCCTGCGTGTAGGCTGGA GCTGCTTC
yihO knockout reverse	TTAATTATTTACAGTAGAAATACTTTGTTTATTATTAGTTCATATGAATATCCT CCTTAG
yihP knockout forward	GAGAAGAATAATGAGTCAAACATCTGTGTAGGCTGGAGCTGCTTC
yihP knockout reverse	GTTATATTTTATTGTTGTTAAACCGTATGCATATGAATATCCTCCTTA
iraP knockout forward	ATGAAAAATCTCATAGCAGAGTTGTTGCTTAAGCTAGCCC GTGTAGGCTGGAGCTGCTTC
iraP knockout reverse	TTAGTGCCGGGGGTGTCTCAGCAACTTTTTTACATATTGG CATATGAATATCCTCCTTAG
rpoS knockout forward	ATGAGTCAGAATACGCTGAAAGTTCATGATTTAAATGAAG GTGTAGGCTGGAGCTGCTTC
rpoS knockout reverse	TTACTCGCGGAACAGCGCTTCGATATTCAGCCCCTGCGTC CATATGAATATCCTCCTTAG
tviA knockout forward	ATGAGGTTTCATCATTTCTGGCCTCCGAATGATATCTATTGTGTAGGCTGGAG CTGCTTC
tviA knockout reverse	TTACAGTAAAGTAACTGAATCCGGCAATAACAGATAGCGCCATATGAATATC CTCCTTAG
tviD knockout forward	ATGAATTTAATGAAATCGTCAGGGATGTTTACGCTTACAGGTGTAGGCTGGA GCTGCTTC
tviD knockout reverse	TTACGACTTCCCTGATGTATTTTTTTGTAATGCGGTTATGCATATGAATATCCT CCTTAG
waaZ knockout forward	ATGGGCAGCGTTAACTTCATAACTCACGCCGATGTTCTGC GTGTAGGCTGGAGCTGCTTC
waaZ knockout reverse	CTAGACAATTTTATCGTAATATTTCATCTTCAAGTTCCGA CATATGAATATCCTCCTTAG
sthC knockout forward	TTGATCGCCGACCCGCGAGAATTCGTCCCTACCAGCAAAT GTGTAGGCTGGAGCTGCTTC
sthC knockout reverse	TCACTGGCATTGCTCGTGTAAGATTTCTACGCCAGAAGCG CATATGAATATCCTCCTTAG
csgA forward	CACCCAACGCTAATACCGTT
csgA reverse	AGGGCTTATGCCCTGTTTTT
csgD forward	CAGCTGTCAGATGTGCGATT
csgD reverse	TCTGCTGCTACAATCCAGGT
yihO forward	TGCGGGGCGTTTTGAGAGGCGA
yihO reverse	TGAATAAAGCGGCAAGCGTCG

bioRxiv preprint doi: https://doi.org/10.1101/2023.10.26.564249; this version posted October 7, 2024. The copyright holder for this preprint (which was not certified by peer review) is the author/funder, who has granted bioRxiv a license to display the preprint in perpetuity. It is made available under aCC-BY 4.0 International license.

yihP forward	GTTGCGATTGGACGCTGTACCTG
yihP reverse	GTAATATGCAGGCATCCCGAGTTC
iraP forward	AGTGATAACGTCACCCTGGAAC
iraP reverse	AGTAACGTTATAACAACTGTGT
rpoS forward	CAGTCTGTCGACTGGCCTTT
rpoS reverse	CTAGTTCCGTCAAGGGATCA
tviA forward	AGGTTATTTCAGCATAAGGA
tviA reverse	TGTCCGTGTTTTACTCAATA
tviD forward	CTCGGTATAACTACTCACTT
tviD reverse	TTCCTAGTGCAGCTAACT
waaZ forward	GACATACTTGAGAGAATTTG
waaZ reverse	TGCGTGCCGAAGCAACGCAA
sthC forward	ACCGGCTGGATTTAGCGATC
sthC reverse	CATGATGCCAGACCCGTGAA
**RT-qPCR**	
rrsA internal forward	GCACCGGCTAACTCCGTGCC
rrsA internal reverse	GCAGTTCCCAGGTTGAGCCCG
rpoS internal forward	CCTGCGTCTGGTGGTAAA
rpoS internal reverse	TTCTCGACTGCACGGATAAG
waaZ internal forward	CCGCTATTCAGGTTGCCTATTC
waaZ internal reverse	AGGGCTGGTAGATTCGTCATAG
sthC internal forward	GATGAAGACGACGATACGGAAG
sthC internal reverse	CGTCTTTCGCGGAGTTCATA
tviB internal forward	TGTGGTAAAGGAACTCGGTAAA
tviB internal reverse	GACTTCCGATACCGGGATAATG
**EMSA**	
Biotin-waaZpro forward	Biotin - GCTGACTGACTTTTATTTGC
waaZpro reverse	ACTCGTATGTTTATCATGCA
waaZpro forward	GCTGACTGACTTTTATTTGC
Biotin-sthCpro forward	Biotin - ACTTCCCCGAGCTTAAAAAT

bioRxiv preprint doi: https://doi.org/10.1101/2023.10.26.564249; this version posted October 7, 2024. The copyright holder for this preprint (which was not certified by peer review) is the author/funder, who has granted bioRxiv a license to display the preprint in perpetuity. It is made available under aCC-BY 4.0 International license.

sthCpro reverse	CGCGATTTGAGGGATCGCTA
sthCpro forward	ACTTCCCCGAGCTTAAAAAT
Biotin-tviApro forward	Biotin - CGTTAGTACTATTAAAATTAGG
tviApro reverse	CTCCTTATGCTGAAATAACCTAA
tviApro forward	CGTTAGTACTATTAAAATTAGG
Biotin-tviBpro	Biotin-GTACGGTTATACGTTTTCAT
tviBpro reverse	CACTCTTATTAATCCTTTACT
