# Supplementary material for: RpoS activates Salmonella Typhi biofilms and drives persistence in a Zebrafish model
Source: bioRxiv. 2023 Oct 26:2023.10.26.564249. Preprint. [Version 1] doi: 10.1101/2023.10.26.564249 (PMC10634867; doi:10.1101/2023.10.26.564249)
Supplement: Supplementary file 1 [file NIHPP2023.10.26.564249V1-supplement-1.pdf]

bioRxiv preprint doi: https://doi.org/10.1101/2023.10.26.564249; this version posted October 26, 2023. The copyright holder for this preprint (which was not certified by peer review) is the author/funder, who has granted bioRxiv a license to display the preprint in perpetuity. It is made available under aCC-BY 4.0 International license.

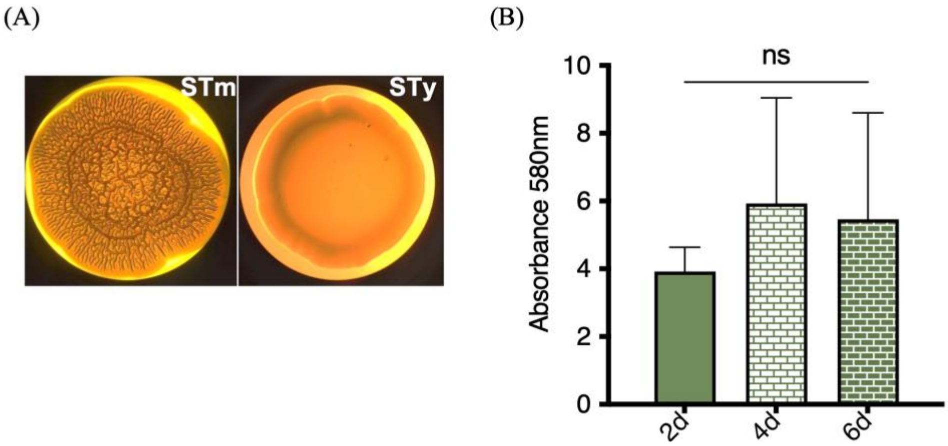

Supplementary Figure 1: H58 forms 'atypical' biofilms. (A) Examination of a macrocolony of *S.* Typhimurium (STm) displaying a red, dry and rough (rdar) morphotype on low salt Luria-Bertani agar medium containing the congo red dye, while *S.* Typhi (STy) are 'smooth' and lack the rdar morphology. (B) The cholesterol-attached biomass formed by wild type H58 did not increase significantly at days 4 and 6 compared to day 2, as determined by crystal violet staining assay. Growth medium added to cholesterol-coated Eppendorf tubes was used as the control and subtracted from all measurements. N = 3, in at least triplicates, error bars represent Mean ± SD, ns = not significant by one-way ANOVA.

bioRxiv preprint doi: https://doi.org/10.1101/2023.10.26.564249; this version posted October 26, 2023. The copyright holder for this preprint (which was not certified by peer review) is the author/funder, who has granted bioRxiv a license to display the preprint in perpetuity. It is made available under aCC-BY 4.0 International license.

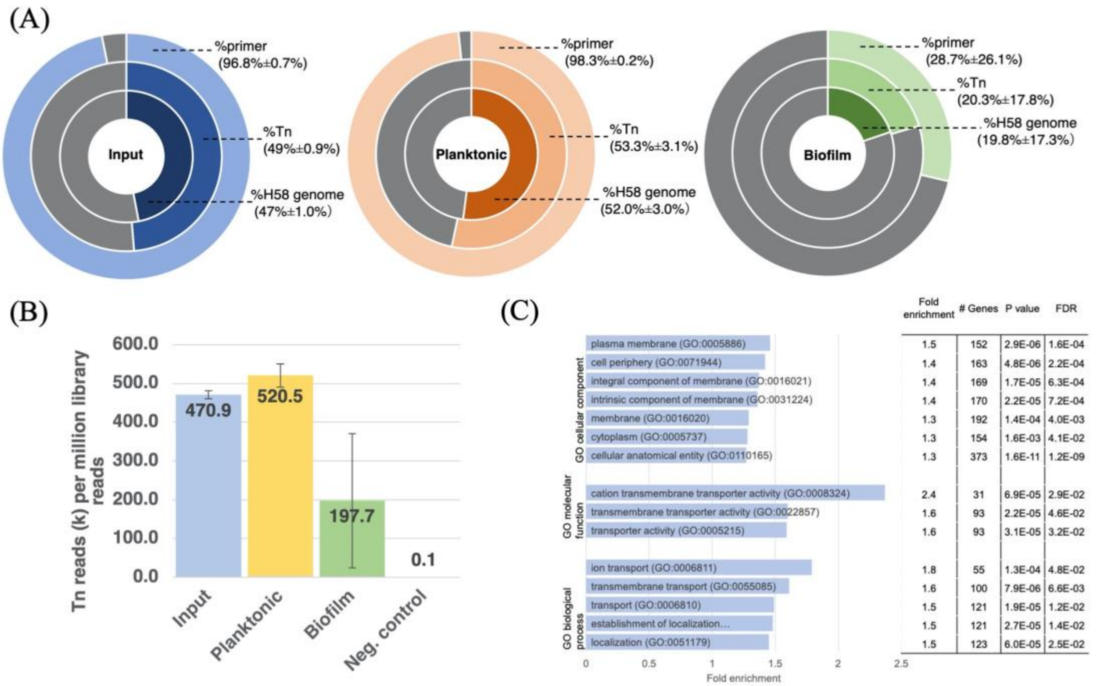

Supplementary Figure 2: (A) Percentages of reads that matched the primer, transposon sequence (IR sequence) and the host genome from left to right: input, planktonic and biofilm sub-populations. (B) Graphical representation of Tn insertion sites per one million of raw reads as obtained by Tn-ClickSeq showing an average of 470,000 reads for the input fraction, 520,000 for the planktonic fraction and 197,000 for the biofilm fraction. A similar analysis using an H58 strain without any transposon insertions resulted in only 100 false positives. (C) A Gene Ontology analysis of transposon insertion sites in 1515 genes that were enriched in the planktonic sub-population as compared to biofilms showed a significant enrichment of cell membrane components, transmembrane ion transport pathways and other membrane related activities.

bioRxiv preprint doi: https://doi.org/10.1101/2023.10.26.564249; this version posted October 26, 2023. The copyright holder for this preprint (which was not certified by peer review) is the author/funder, who has granted bioRxiv a license to display the preprint in perpetuity. It is made available under aCC-BY 4.0 International license.

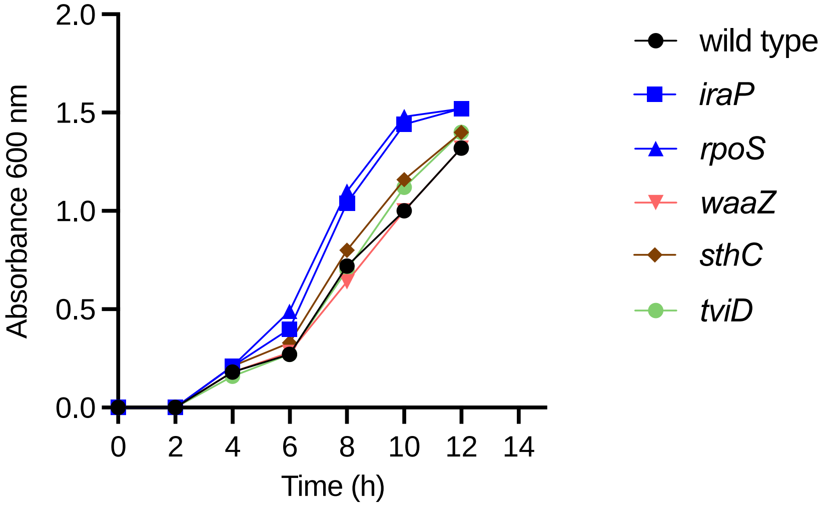

Supplementary Figure 3: Biofilm mutants are not compromised in planktonic growth. Strains deleted of *iraP*, *rpoS*, *waaZ*, *sthC* or *tviD* and the wild type H58 strain were grown for 12 hours in Luria-Bertani broth at 37°C/250 rpm and the absorbance at 600 nm was measured every 2 hours.

bioRxiv preprint doi: https://doi.org/10.1101/2023.10.26.564249; this version posted October 26, 2023. The copyright holder for this preprint (which was not certified by peer review) is the author/funder, who has granted bioRxiv a license to display the preprint in perpetuity. It is made available under aCC-BY 4.0 International license.

Supplementary Table 1: List of bacterial strains and plasmids

Strain or plasmid	Description	Source or Reference
14028s	*Salmonella enterica* serovar Typhimurium wild type strain	Lab collection
DW85	14028s (*ssrB*::*kan*)	Lab collection
H58 ('wild type' in this study)	*Salmonella enterica* serovar Typhi wild type strain	Stephen Baker, Cambridge University, UK
Ty2b	*Salmonella enterica* serovar Typhi wild type strain	Salmonella Genetic Stock Center, University of Calgary, CA
CT18	*Salmonella enterica* serovar Typhi wild type strain	Salmonella Genetic Stock Center, University of Calgary, CA
ct117	*Salmonella enterica* serovar Typhi wild type strain	Salmonella Genetic Stock Center, University of Calgary, CA
ssrB	H58 (*ssrB*::*kan*)	This study
csgD	H58 (*csgD*::*cat*)	This study
csgA	H58 (*csgA*::*kan*)	This study
yihO	H58 (*yihO*::*kan*)	This study
yihP	H58 (*yihP*::*kan*)	This study
yihO yihP	H58 (Δ*yihO yihP*::*kan*)	This study
iraP	H58 (*iraP*::*kan*)	This study
rpoS	H58 (*rpoS*::*kan*)	This study
sthC	H58 (*sthC*::*kan*)	This study
waaZ	H58 (*waaZ*::*kan*)	This study
tviA	H58 (*tviA*::*cat*)	This study
tviB	H58 (*tviD*::*kan*)	This study
rpoSc	H58 (*rpoS*::*kan*, pBR322::*rpoS*)	This study
pBR322::*rpoS* plasmid	*rpoS* cloned between EcoRI and ScaI sites in pBR322	Roy Curtiss III, University of Florida, USA
pFPV::mCherry plasmid	mCherry cloned between XbaI and SphI sites in pFPV	Olivia Steele-Mortimer (Addgene plasmid # 20956 http://n2t.net/addgene:20956; RRID:Addgene_20956

Supplementary Table 2: List of oligonucleotides

Primers	Sequence
**Tn-ClickSeq**	
3'21-39	GTGACTGGAGTTCAGACGTGTGCTCTTCCGATCTTAATACGACTCACTATAGG<br>[Black = Illumina p7 adapter, red = Tn-specific]
**Gene deletions**	

bioRxiv preprint doi: https://doi.org/10.1101/2023.10.26.564249; this version posted October 26, 2023. The copyright holder for this preprint (which was not certified by peer review) is the author/funder, who has granted bioRxiv a license to display the preprint in perpetuity. It is made available under aCC-BY 4.0 International license.

csgD knockout forward	GCAGCTGTCAGATGTGCGATTAAAAAAAGTGGAGTTTCATCATGT TTAATGTGTAGGCTGTAGCTGCTTC
csgD knockout reverse	CTCTGCTGCTACAATCCAGGTCAGATAGCGTTTCATGGCCTTACCG CCTGCCTCCTTAGTTCCTATTCCG
csgA knockout forward	CACCCAACGCTAATACCGTTACGACTTTTAAATCAATCAATCCGGT GTAGGCTGGAGCTGCTTC
csgA knockout reverse	AGGGCTTATGCCCTGTTTTTTTATTAGCGCAGACGCTAAACATATG AATATCCTCCTTAG
yihO knockout forward	ATGTCTAATCATGATCCGCTAACGCTAAAGTTGAGCCTGCGTGTAG GCTGGAGCTGCTTC
yihO knockout reverse	TTAATTATTTACAGTAGAAATACTTTGTTTATTATTAGTTCATATGA ATATCCTCCTTAG
yihP knockout forward	GAGAAGAATAATGAGTCAAACATCTGTGTAGGCTGGAGCTGCTTC
yihP knockout reverse	GTTATATTTTATTGTTGTTAAACCGTATGCATATGAATATCCTCCTT A
iraP knockout forward	ATGAAAAATCTCATAGCAGAGTTGTTGCTTAAGCTAGCCC GTGTAGGCTGGAGCTGCTTC
iraP knockout reverse	TTAGTGCCGGGGGTGTCTCAGCAACTTTTTTACATATTGG CATATGAATATCCTCCTTAG
rpoS knockout forward	ATGAGTCAGAATACGCTGAAAGTTCATGATTTAAATGAAG GTGTAGGCTGGAGCTGCTTC
rpoS knockout reverse	TTACTCGCGGAACAGCGCTTCGATATTCAGCCCCTGCGTC CATATGAATATCCTCCTTAG
tviA knockout forward	ATGAGGTTTCATCATTTCTGGCCTCCGAATGATATCTATTGTGTAG GCTGGAGCTGCTTC
tviA knockout reverse	TTACAGTAAAGTAACTGAATCCGGCAATAACAGATAGCGCCATAT GAATATCCTCCTTAG
tviD knockout forward	ATGAATTTAATGAAATCGTCAGGGATGTTTACGCTTACAGGTGTAG GCTGGAGCTGCTTC
tviD knockout reverse	TTACGACTTCCCTGATGTATTTTTTTGTAATGCGGTTATGCATATGA ATATCCTCCTTAG

bioRxiv preprint doi: https://doi.org/10.1101/2023.10.26.564249; this version posted October 26, 2023. The copyright holder for this preprint (which was not certified by peer review) is the author/funder, who has granted bioRxiv a license to display the preprint in perpetuity. It is made available under aCC-BY 4.0 International license.

waaZ knockout forward	ATGGGCAGCGTTAACTTCATAACTCACGCCGATGTTCTGC GTGTAGGCTGGAGCTGCTTC
waaZ knockout reverse	CTAGACAATTTTATCGTAATATTTCATCTTCAAGTTCCGA CATATGAATATCCTCCTTAG
sthC knockout forward	TTGATCGCCGACCCGCGAGAATTCGTCCCTACCAGCAAAT GTGTAGGCTGGAGCTGCTTC
sthC knockout reverse	TCACTGGCATTGCTCGTGTAAGATTTCTACGCCAGAAGCG CATATGAATATCCTCCTTAG
csgA forward	CACCCAACGCTAATACCGTT
csgA reverse	AGGGCTTATGCCCTGTTTTT
csgD forward	CAGCTGTCAGATGTGCGATT
csgD reverse	TCTGCTGCTACAATCCAGGT
yihO forward	TGCGGGGCGTTTTGAGAGGCGA
yihO reverse	TGAATAAAGCGGCAAGCGTCG
yihP forward	GTTGCGATTGGACGCTGTACCTG
yihP reverse	GTAATATGCAGGCATCCCGAGTTC
iraP forward	AGTGATAACGTCACCCTGGAAC
iraP reverse	AGTAACGTTATAACAACTGTGT
rpoS forward	CAGTCTGTCGACTGGCCTTT
rpoS reverse	CTAGTTCCGTCAAGGGATCA
tviA forward	AGGTTATTTCAGCATAAGGA
tviA reverse	TGTCCGTGTTTTACTCAATA
tviD forward	CTCGGTATAACTACTCACTT
tviD reverse	TTCCTAGTGCAGCTAACT
waaZ forward	GACATACTTGAGAGAATTTG
waaZ reverse	TGCGTGCCGAAGCAACGCAA
sthC forward	ACCGGCTGGATTTAGCGATC

bioRxiv preprint doi: https://doi.org/10.1101/2023.10.26.564249; this version posted October 26, 2023. The copyright holder for this preprint (which was not certified by peer review) is the author/funder, who has granted bioRxiv a license to display the preprint in perpetuity. It is made available under aCC-BY 4.0 International license.

sthC reverse	CATGATGCCAGACCCGTGAA
**RT-qPCR**	
rrsA internal forward	GCACCGGCTAACTCCGTGCC
rrsA internal reverse	GCAGTTCCCAGGTTGAGCCCG
rpoS internal forward	CCTGCGTCTGGTGGTAAA
rpoS internal reverse	TTCTCGACTGCACGGATAAG
waaZ internal forward	CCGCTATTCAGGTTGCCTATTC
waaZ internal reverse	AGGGCTGGTAGATTCGTCATAG
sthC internal forward	GATGAAGACGACGATACGGAAG
sthC internal reverse	CGTCTTTCGCGGAGTTCATA
tviB internal forward	TGTGGTAAAGGAACTCGGTAAA
tviB internal reverse	GACTTCCGATACCGGGATAATG
